# Circulating Tumor DNA Monitoring Reveals Molecular Progression before Radiologic Progression in a Real-life Cohort of Patients with Advanced Non–small Cell Lung Cancer

**DOI:** 10.1158/2767-9764.CRC-22-0258

**Published:** 2022-10-13

**Authors:** Malene S. Frank, Christina S.A. Andersen, Lise B. Ahlborn, Niels Pallisgaard, Uffe Bodtger, Julie Gehl

**Affiliations:** 1 Department of Clinical Oncology and Palliative Care, Zealand University Hospital, Copenhagen, Denmark.; 2 Department of Clinical Medicine, Faculty of Health and Medical Sciences, University of Copenhagen, Copenhagen, Denmark.; 3 Department of Pathology, Zealand University Hospital Næstved, Denmark.; 4 Department of Science and Environment, Roskilde University, Denmark.; 5 Center for Genomic Medicine, Rigshospitalet, Copenhagen, Denmark.; 6 Department of Respiratory Medicine, Zealand University Hospital, Næstved, Denmark.; 7 Institute for Regional Health Research, University of Southern Denmark, Denmark.

## Abstract

**Purpose:**

The clinical potential of liquid biopsy in patients with advanced cancer is real-time monitoring for early detection of treatment failure. Our study aimed to investigate the clinical validity of circulating tumor DNA (ctDNA) treatment monitoring in a real-life cohort of patients with advanced non–small cell lung cancer (NSCLC).

**Experimental Design:**

Patients with advanced or noncurative locally advanced NSCLC were prospectively included in an exploratory study (NCT03512847). Selected cancer-specific mutations were measured in plasma by standard or uniquely designed droplet digital PCR assays before every treatment cycle during first-line treatment until progressive disease (PD). Correlation between an increase in ctDNA (= molecular progression) and radiologic PD was investigated, defined as lead time, and the corresponding numbers of likely futile treatment cycles were determined. Utility of ctDNA measurements in clarifying the results of nonconclusive radiologic evaluation scans was evaluated.

**Results:**

Cancer-specific mutations and longitudinal plasma sampling were present in 132 of 150 patients. ctDNA was detectable in 88 (67%) of 132 patients treated by respectively chemotherapy (*n* = 41), immunotherapy (*n* = 43), or combination treatment (*n* = 4). In 66 (90%) of 73 patients experiencing PD, a ctDNA increase was observed with a median lead time of 1.5 months before radiologic PD. Overall, 119 (33%) of 365 treatment cycles were administered after molecular progression. In addition, ctDNA measurements could clarify the results in 38 (79%) of 48 nonconclusive radiologic evaluations.

**Conclusions:**

ctDNA monitoring leads to earlier detection of treatment failure, and clarifies the majority of nonconclusive radiologic evaluations, giving the potential of sparing patients from likely futile treatments and needless adverse events.

**Significance:**

Treatment monitoring by ctDNA has the clinical potential to reveal PD before radiologic evaluation and consequently spare patients with advanced cancer from likely ineffective, costly cancer treatments and adverse events.

## Introduction

Lung cancer is the leading cause of cancer-related death worldwide. Non–small cell lung cancer (NSCLC) is the most common subtype, and approximately half of patients are diagnosed with advanced disease with the only treatment option being life-prolonging treatment (1). Despite decades of advances in precision medicine and latest the implementation of immunotherapy in treatment guidelines, the 5-year survival rate is of only 6% (2, 3). Performance status (PS) deterioration due to progressive symptoms and adverse events by systemic treatments are major challenges in the management of patients with advanced NSCLC (4, 5). In addition, response rates are low in general, varying from 15% to 45% in immunotherapy-treated patients (6), and 25% in chemotherapy-treated patients (7), and treatment options are limited.

The precision of conventionally radiologic evaluation scans have been challenged by immunotherapy-induced recruitment of immune cells resembling increment in tumor size, named “pseudoprogression.” Thus, an urgent need for a more precise and reliable method of treatment monitoring is needed to reduce ineffective treatments and consequent needless adverse events.

Liquid biopsy has the potential to overcome these challenges by measuring molecular changes with high precision, distinguishing circulating tumor DNA (ctDNA) from normal DNA in a dynamic manner in consecutive plasma samples. Recent explorative studies have demonstrated its promising potential as a biomarker predictive of treatment efficacy (8–23) and overall survival (OS; refs. 8, 9, 12, 14–19, 22, 23). The majority of ctDNA research in NSCLC has been focusing on selected patient groups, for example, with specific driver mutations (11, 14, 24–26), and/or with a limited number of longitudinal blood samples available (8–22, 24, 27). Other previous studies have reported on heterogeneous cohorts of patients with different cancers and various different treatment lines (8, 12, 17, 23), and/or a limited number of patients (10, 12–14, 19, 21, 28).

Moreover, a variety of different methods of collecting and processing plasma samples, and detecting and reporting ctDNA, have been explored, highlighting the need of standardization, focusing on clinical implementation and feasibility (29, 30).

Thus, the linkage to clinical practice, representing “real-life patients,” and “real-time” analyses is required to move from clinical validity to clinical utility.

In this study, we prospectively included and followed a cohort of patients with unselected advanced or noncurative locally advanced NSCLC from first-line treatment initiation to progressive disease (PD), concurrently measuring the level of ctDNA for correlation to treatment outcome—including comprehensive clinical measurements, like hospitalizations, adverse events, PS changes, and second-line treatment.

## Materials and Methods

### Aims

The primary aim of the study was to investigate the clinical validity of ctDNA measurements during first-line treatment in advanced NSCLC by reporting on the correlation between ctDNA dynamics and treatment efficacy by radiologic evaluation. More precisely, we intended to measure the lead time (days) from a substantial ctDNA increase (= molecular progression) to radiologically verified progression. The secondary aims were reporting on the number of treatments given beyond ctDNA progression including adverse events and number of hospitalizations, as well as PS changes and second-line treatment.

### Study Design

The study is an explorative single centre study (Clinicaltrials.gov: NCT03512847), conducted at Department of Clinical Oncology and Palliative Care, Zealand University Hospital. It was approved by The Regional Committee on Health Research Ethics (SJ-662) and The Danish Data Protection Agency (REG-006-2018), and was conducted according to the Helsinki Declaration with written informed consent from all included patients, and following the STROBE (STrengthening the Reporting of OBservational studies in Epidemiology) guidelines.

### Patients

Patients with newly diagnosed advanced or noncurative locally advanced NSCLC without actionable *EGFR* mutations or *ALK* rearrangements were screened for eligibility in the period from June 29, 2018 to November 1, 2020. Inclusion criteria were: Age >18 years, Eastern Cooperative Oncology Group (ECOG) score of PS 0–2, measurable disease according to the RECIST, version 1.1, and ability to understand spoken and written Danish. Exclusion criteria were: Other active cancers and contraindications for systemic treatment. The study cohort has previously been described in two publications, reporting on feasibility of rebiopsy including programmed death-ligand 1 (PD-L1) changes (31) and actionable molecular alterations (32).

### Treatment and Evaluation

Patients were treated according to national treatment guidelines (33), depending on pathology, PD-L1 tumor proportion score, ECOG PS, and renal function and/or comorbidity status. Treatments included immunotherapy (pembrolizumab), chemotherapy (carboplatin or cisplatin and vinorelbine, or monotherapy vinorelbine), or combination treatment (carboplatin/cisplatin, pemetrexed, and pembrolizumab, followed by maintenance pemetrexed/pembrolizumab). Details of treatment are described in ref. 32.

Evaluation of treatment efficacy was performed by CT scans after every second or third treatment cycle through the RECIST or immunotherapy-related RECIST (iRECIST), depending on treatment type.

### Tissue and Blood Sampling

**FIGURE 1:**
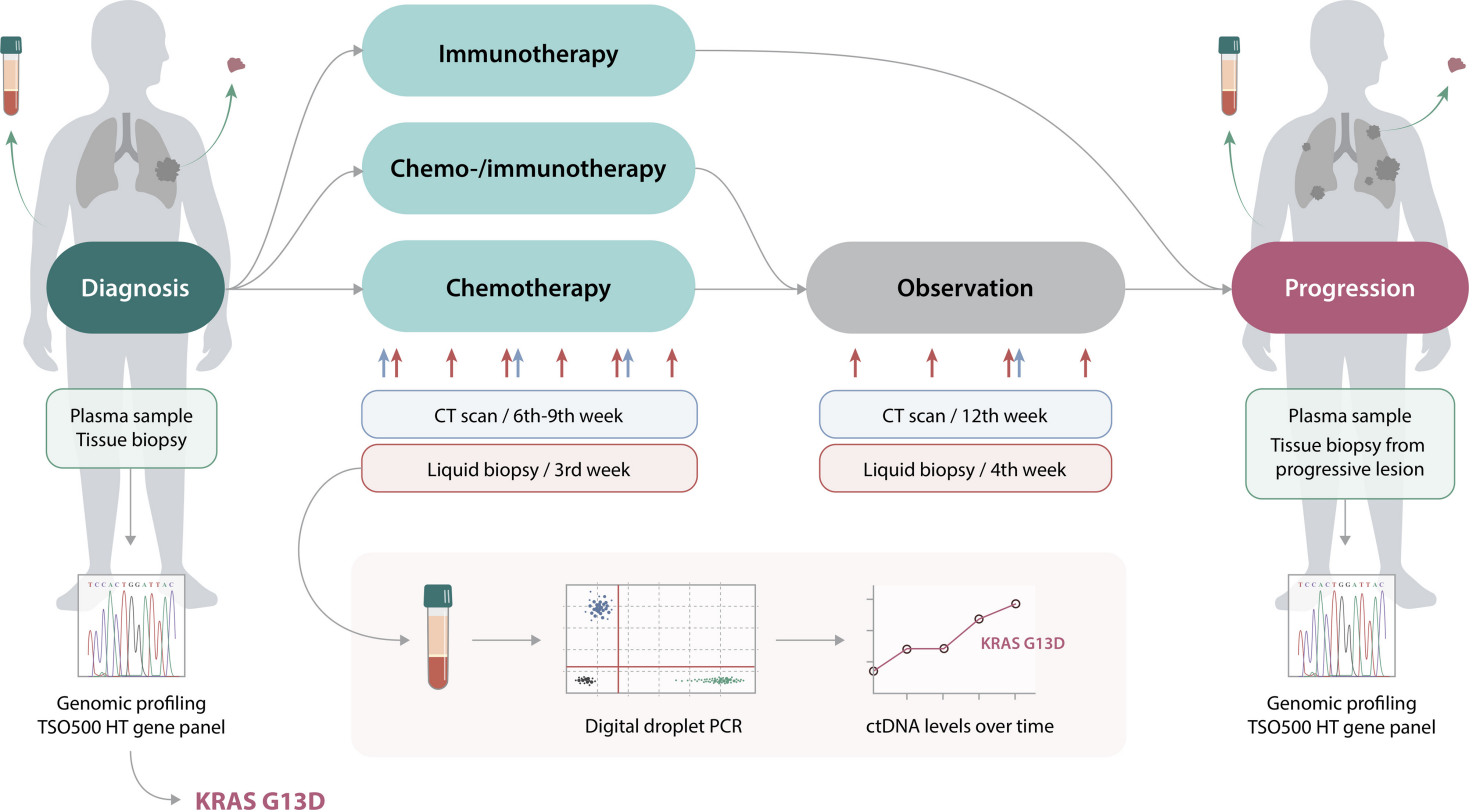
Study design.

Blood samples were collected at diagnosis and before every treatment cycle (every third week) until PD, defined by CT scan (Fig. 1). Patients with stable disease (SD) or regression after completion of systemic treatment were proposed a continuation of blood sampling every fourth week. All patients had a tissue biopsy taken at time of diagnosis—and at time of progression, if feasible and accepted by the patient. Details of rebiopsy modalities, locations, and complications are described in a previous publication (31).

### Tissue DNA Sequencing and Mutational Analyses

All tissue biopsies were sequenced by using the TruSight Oncology (TSO) 500 HT gene panel (Illumina). Descriptions of DNA extraction, sequencing, and interpretation are available in ref. 32. Sequencing data including likely pathogenic and pathogenic somatic mutations including nonsense, frameshift, missense, and splice site alterations in cancer-related genes, are available in Supplementary Table S1, which also details location of biopsies, microsatellite instability, and tumor mutational burden.

### Plasma and Cell-free DNA Isolation

Peripheral blood samples (50 mL) were collected in Ethylenediaminetetraacetic acid (EDTA) tubes and processed within 2 hours. Plasma was isolated by centrifugation 2,000 × *g* for 10 minutes at 20°C, retrieving the plasma supernatant avoiding buffy coat contamination and performing a second centrifugation at 10,000 × *g* for 10 minutes at 20°C. After plasma isolation, a 191 bp *CPP1* DNA spike-in control fragment was added and the samples were stored at −80°C until DNA extraction (34). Cell-free DNA (cfDNA) was purified from 3 to 5 mL plasma on a Perkin Elmer Chemagic 360 robot using a CMG1304 kit according to the manufacturer's recommendations and eluted in 100 μL eluat. Quality control of each purified DNA was performed by measuring the amount of spike-in *CPP1* DNA and the amount of potential contaminating lymphocyte DNA measured by immunoglobulin gene rearrangements using a multiplex droplet digital PCR (ddPCR). Overall, we observed in majority of samples a low grade (below 0.2%) of lymphocyte DNA contamination, in 14% a medium grade (0.2%–0.99%), and in only four samples a contamination above 1% (range, 1%–5%). In addition, the degree of DNA fragmentation was measured by a multiplex ddPCR assay detecting a 65 bp and a 250 bp fragment of the *EMC7* housekeeping gene (34, 35). The *EMC7* 65 bp assay was used to quantify the amount of sample cfDNA.

### ctDNA Analyses

ctDNA was analyzed by ddPCR for tumor specific mutations, identified in tissue biopsies. We selected mutations previously identified in cancers (Catalogue of Somatic Mutations in Cancer, COSMIC), relevant databases (e.g.*,* CKB-BOOST, ClinVar), or mutations previously described in the literature. One mutation was selected in each patient case, based on (i) the cancer relevance (COSMIC, relevant databases etc.), (ii) the availability of standard assays (Supplementary Table S2), and/or (iii) the highest allele frequency. If no standard assay was available, a patient specific assay was designed and validated on DNA from the tissue biopsies. All plasma samples for each patient case were analyzed to monitor changes in ctDNA levels during treatment and follow-up.

The ddPCR analyses were conducted in duplicates using 5 μL template DNA, 300 nmol/L primers, 200 nmol/L probe, 2x Supermix for Probes (no dUTP) (Bio-Rad), and Ambion Nuclease-Free Water (Invitrogen) with a final volume of 20 μL per reaction. Bio-Rad Automated Droplet Generator (Bio-Rad) was used for droplet generation, a Veriti 96-well Thermocycler (Applied Biosystems) for PCR amplification, and a Bio-Rad QX200 Droplet Reader. PCR cycling conditions were as recommended by Bio-Rad. A negative control (water), a wild-type control (blood DNA), and a positive mutation control (DNA from tissue biopsy) were included in each ddPCR analysis and the results were analyzed using QuantaSoft Software (Bio-Rad) version 1.4.7. A minimum of 10,000 total droplets was required for each well to be included in the analyses. Samples were normalized to the median fluorescence of the negative population, the wild-type control and the positive control. The threshold for calling a sample positive was set to ≥3 positive droplets. The results were calculated and reported as copies per μL plasma with a 95% Confidence Interval (CI) based on Poisson distribution. For samples under the level of detection (≤2 positive droplets), a mean CI was calculated based on 20 negative samples.

Assays detecting nucleotide changes, which were not commercially available from Bio-Rad, were either ordered as customer-designed assays from Bio-Rad or designed as in-house assays using the Oligo 7 Software, version 7.60 and ordered from LGC Biosearch Technologies. For assay validation, primers were designed to amplify 180–220 bp fragments containing the target of interest using DNA extracted from the patient's diagnostic tissue samples as a template. Primer sequences are available in Supplementary Table S2.

### Statistical Analysis

Descriptive statistics were applied for clinical, pathologic, and molecular characteristics and presented as frequencies, percentages, median (range), and mean (95% CI). Progression-free survival (PFS) was defined as the time from first-line treatment initiation to radiologically verified PD or until death of any cause. Patients without PD by the cut-off date of March 1, 2022 were listed. OS was defined as the time from first-line treatment initiation to death of any cause or until the cut-off date. PFS and OS were calculated by the Kaplan–Meier method and comparisons by log-rank (Mantel–Cox) test.

Independence between different variables and nondetectable/detectable ctDNA was analyzed through Fisher exact test. Variables with confirmed *P* values below 0.15 were included in logistic regression analyses with a significance level of *P* < 0.05. Statistical analyses were performed by IBM SPSS Statistics, version 28.0.0.0 and GraphPad prism version 8.4.3.

### Data Management and Definitions

Molecular progression was defined as an increase in ctDNA at any time during treatment or follow-up. The definition of a substantial increase is detailed in Results. The lead time was calculated (in days) from a ctDNA increase (molecular progression) to radiologically verified progression. Fold changes in ctDNA were defined as the difference between the ctDNA increase (*a*) and the preceding value (*b*) divided by the preceding value, calculated by (*a* − *b*)/*b*.

We defined number of hospitalizations as all hospitalizations including admittance to hospice from the beginning of first-line treatment until beginning of second-line treatment or death, but no later than 1 year after ending first-line treatment. Hospitalizations without any obvious relation to adverse events or cancer-related complications were excluded. Adverse events were ranged according to Common Terminology Criteria for Adverse Events (CTCAE) in categories of grade 0–1, 2, or 3–5.

### Data Availability

Raw data (comprehensive sequencing data files) were generated at Center for Genomic Medicine, Rigshospitalet, Copenhagen, Denmark, and derived data are available within the article and its Supplementary Data (Supplementary Table S1).

## Results

### Patient Characteristics

A total number of 150 patients were included from June 29, 2018 to November 1, 2020.

**FIGURE 2:**
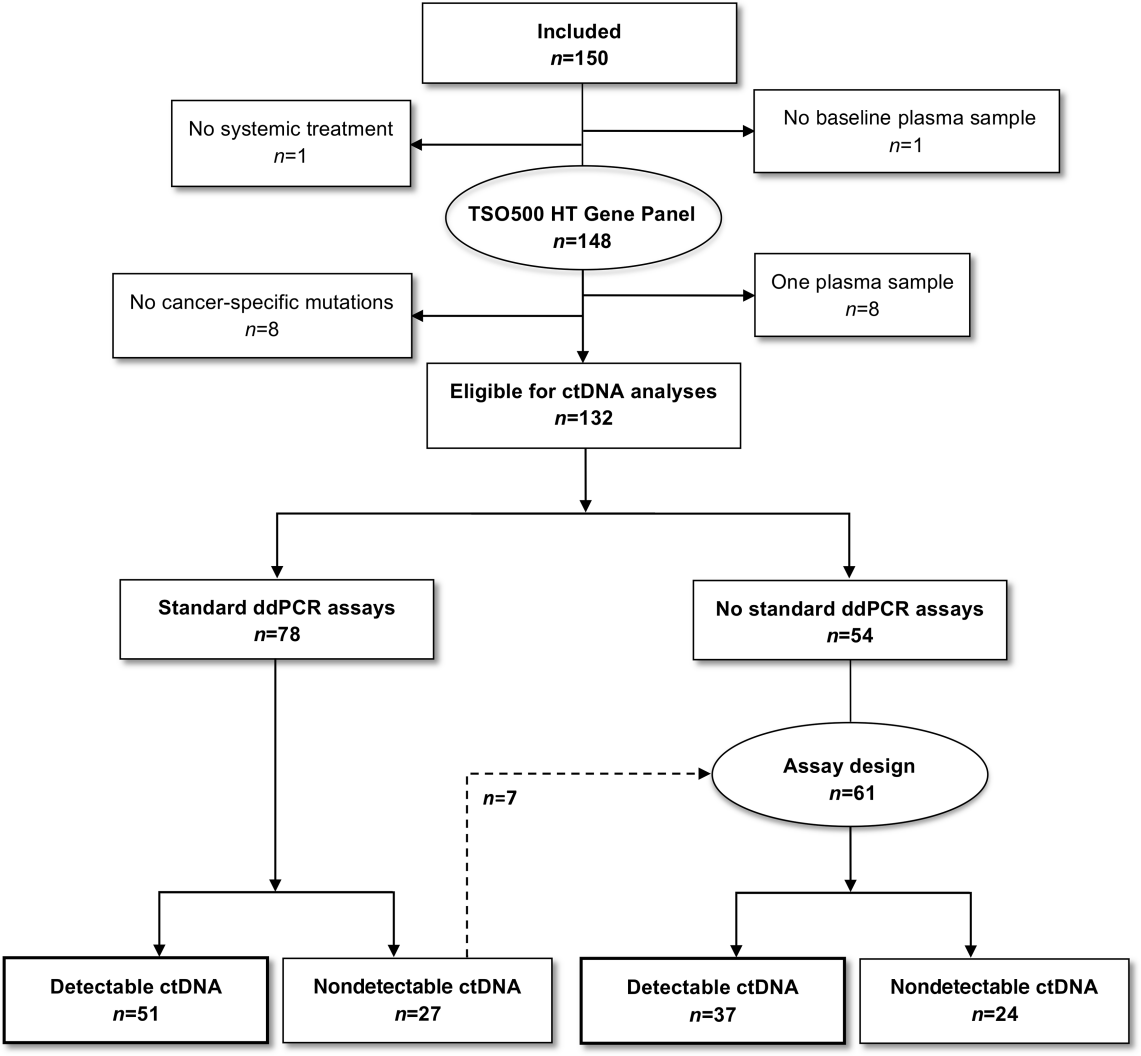
Consort Diagram.

Of the 150 included patients, a total of 148 patients had tissue mutational analyses performed by TSO500 HT gene panel, and 132 patients had ctDNA analyses performed (Fig. 2). Baseline characteristics of the 132 patients having ctDNA analyses performed are detailed in Table 1 and the representativeness in Supplementary Table S3. By the cut-off date of March 1, 2022, 111 patients had radiologically verified progression with a median PFS of 150 days (range, 12–1,209+; Supplementary Fig. S1). Patients received immunotherapy (*n* = 68), chemotherapy (*n* = 57), and combination treatment (*n* = 7), respectively. A total number of 1,392 blood samples were taken with a median of 7 samples (range, 2–43) per patient.

**TABLE 1:** Baseline characteristics of patients eligible for ctDNA analyses (*n* = 132)

Baseline characteristics of all patients (*n* = 132)		
**Sex, *n* (%)** Male Female	64 (48) 68 (52)	
**Age, years, median (range)**	66 (44–84)	
**ECOG Performance score (PS),** ***n* (%)—at time of diagnosis** PS 0 PS 1 PS 2 PS 3	58 (44) 57 (43) 17 (13) 0 (0)	
**Smoking status, *n* (%)** Never Former Current	5 (4) 86 (65) 40 (30)	
NA	1 (1)	
**Histology, *n* (%)** Adenocarcinoma Squamous cell carcinoma Not otherwise specified (NOS)	95 (72) 28 (21) 9 (7)	
**PD-L1 tumor proportion score, *n* (%)** <1% ≥1% <5% ≥5% <50% ≥50% NA	36 (27) 4 (3) 17 (13) 74 (56) 1 (1)	
**Stage, IASCL, 8th edition, *n* (%)** IIIA IIIB IIIC IVA IVB	3 (2) 10 (8) 10 (8) 60 (45) 49 (37)	
**Treatment type, *n* (%)** Cisplatin/carboplatin/vinorelbine (± maintenance pemetrexed) Vinorelbine monotherapy Pembrolizumab Carboplatin/pemetrexed/pembrolizumab	52 (39) 5 (4) 68 (52) 7 (5)	**No. of cycles** **(range)** 4 (1–10) 2 (1–6) 8 (1–36) 7 (4–16)

Abbreviations: ECOG, Eastern Cooperative Oncology Group; IASCL, The International Association for the Study of Lung Cancer; NA, not available; OS, overall survival; PD-L1, programmed death-ligand 1; PFS, progression-free survival.

### Mutations and Assays

**FIGURE 3:**
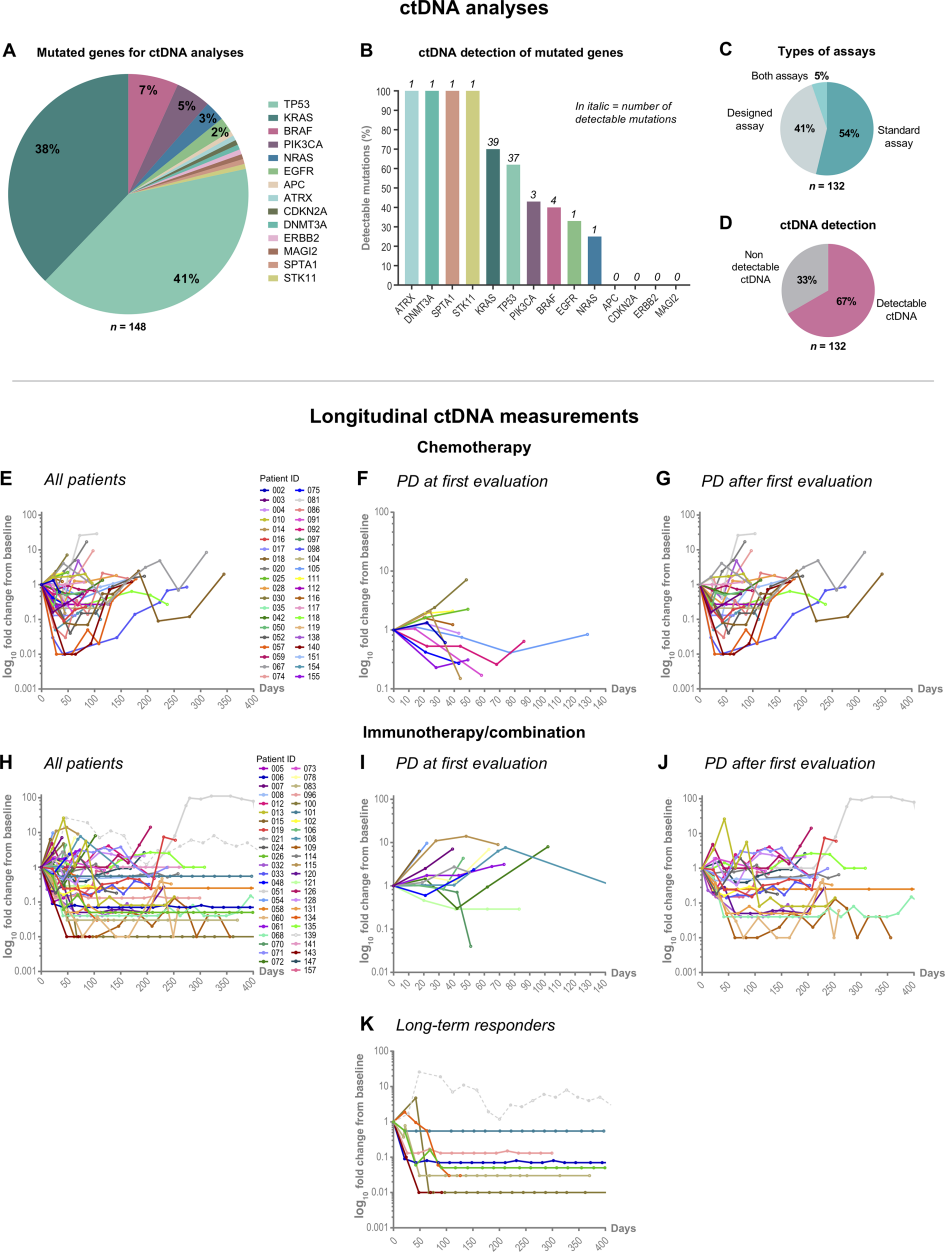
ctDNA analyses and longitudinal measurements. **A,** illustrates the frequencies of the mutated genes applicable for ctDNA analyses. **B,** depicts the detection of ctDNA (%) of each mutated gene. **C,** shows the distribution of patients having ctDNA analyses performed by standard assays, designed assays, and both. **D,** illustrates the distribution of patients with detectable (pink) and nondetectable (gray) ctDNA. **E–K,** illustrates the fold changes over time (log_10_ scale) related to the baseline level in chemotherapy-treated patients (**E–G)** and immunotherapy/combination-treated patients (**H–K**)—divided into treatment response. Gray dashed line in **K** depicts one patient with suspected measurements of a subclonal mutation. PD = progressive disease.

A total of 148 mutations in 14 different genes were revealed, including *TP53* (41%, *n* = 60), *KRAS* (38%, *n* = 56), *BRAF* (7%, *n* = 10) *PIK3CA* (5%, *n* = 7), *NRAS* (3%, *n* = 4), *EGFR* (2%, *n* = 3)—being the most frequent—as illustrated in Fig. 3A. Details of transcript and protein variants, and allele fractions are depicted in Supplementary Table S1. Analyses of ctDNA were performed by standard assays in 54% (*n* = 71) and variant-specific designed assays in 41% (*n* = 54) of cases (Fig. 3C).

### ctDNA Detection

Overall, we found detectable ctDNA in 67% (*n* = 88) of patients (Fig. 3D). The detection rate for standard assays and designed assays was 65% and 61%, respectively. Figure 3B illustrates the detection of ctDNA of each specific gene. In 5% (*n* = 7) of patients, we performed standard assay analyses (*BRAF G469, BRAF V600E, KRAS G12A, KRAS G12R, KRAS G13C, KRAS G13D, PIK3CA H1047R*) showing no detectable ctDNA, and to clarify if other mutations could be detectable, we additionally designed assays (*TP53* variants) matching other identified mutations in each patient case. Detectable ctDNA was found by a designed assay in only one of these patient cases, indicating that other factors, than choosing the right cancer-specific mutation, affects the detectability. As suggested by others the shedding of ctDNA can be related to, for example*,* specific clinicopathologic characteristics (36–39). To investigate whether certain characteristics were related to having detectable ctDNA, we performed *χ*^2^/Fisher exact test and regression analyses and found that adenocarcinoma histology increases the odds almost 4-fold (3.868; 95% CI, 1.421–10.525) of having detectable ctDNA, while presence of adrenal gland metastases increases the odds 2.5-fold (2.515; 95% CI, 1.007–6.284). Supplementary Table S4 details the results including all investigated variables.

### Baseline cfDNA/ctDNA Correlation to OS and PFS

A significant difference in OS was observed in patients with detectable versus nondetectable ctDNA (median 296 vs. 578 days; HR, 0.66; 95% CI, 0.44–0.98; *P* = 0.05; Supplementary Fig. S2) and between patients having a ctDNA baseline level above/below the median level (285 vs. 414 days; HR, 0.61; 95% CI, 0.37–0.99; *P* = 0.04; Supplementary Fig. S3). No significant differences in PFS or in cfDNA correlation to OS/PFS were observed (Supplementary Fig. S4).

### Longitudinal ctDNA Measurements

#### Molecular Progression

Figure 3E–K illustrates fold changes over time related to the baseline level in patients with longitudinally detectable ctDNA (*n* = 87). In one chemotherapy-treated patient, the longitudinal ctDNA measurements were not interpretable due to the assay quality. In one patient experiencing long-term response of immunotherapy, we suspected measurements of a subclonal mutation due to continuously varying ctDNA measurements during 2 years of treatment with no evidence of radiologic PD (Fig. 3K). To determine whether the mutation could be clonal hematopoiesis of indeterminate potential (CHIP; ref. 40), we analyzed buffy coat and found no presence of the mutation.

**FIGURE 4:**
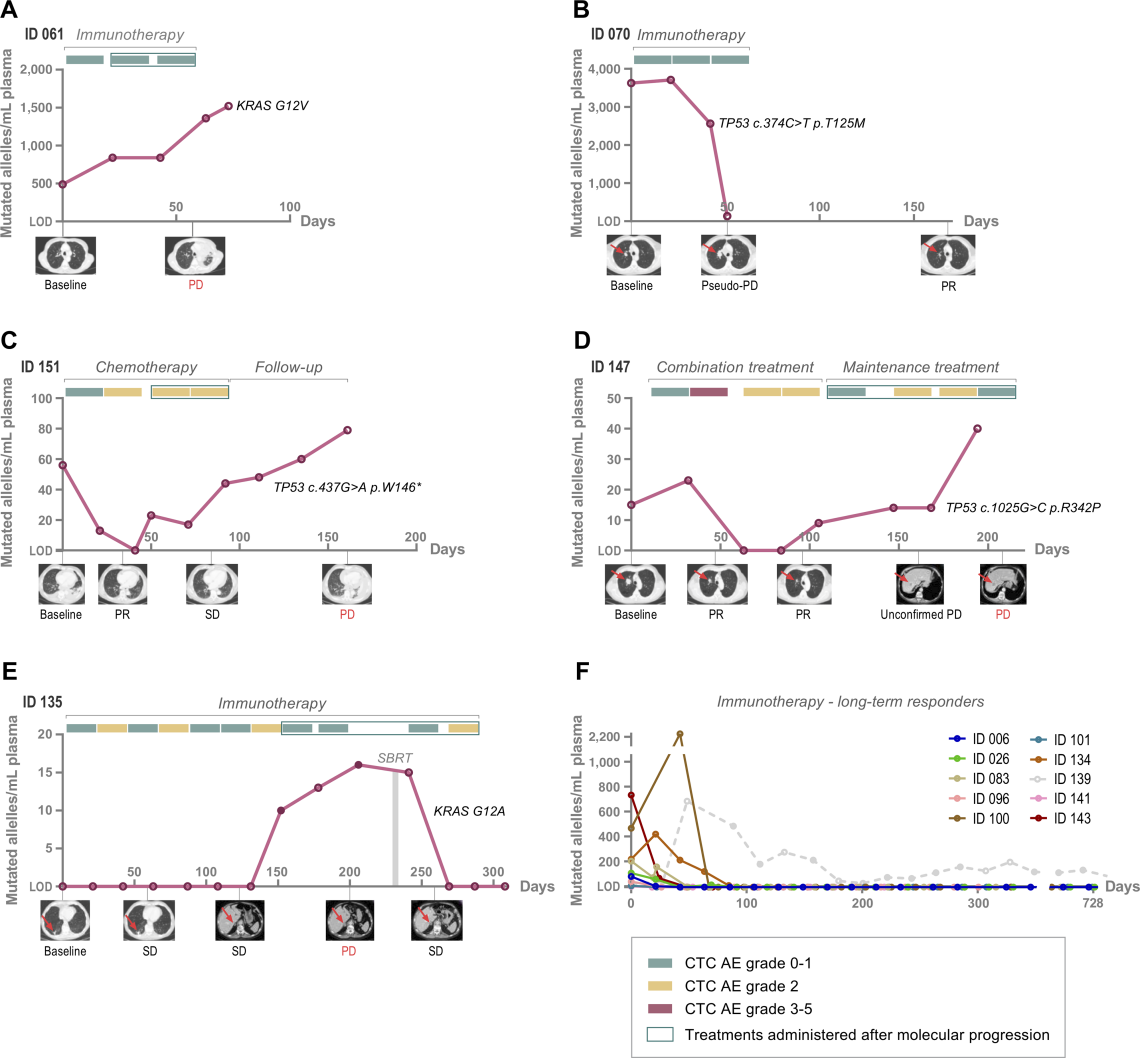
Patient cases. **A,** A 63-year-old woman treated by immunotherapy, experiencing radiologic PD after three treatment cycles (TCs), but persisting molecular progression (= ctDNA increase) after the first TC. **B,** A 66-year-old male treated by immunotherapy with radiologic PD, after three TCs, but no subjective symptoms or objective signs of PD. Subsequently, radiologic PR was observed with a significant decrease in the size of tumor/metastases. Pseudoprogression after first-line immunotherapy was suspected supported by an early and persistent drop in ctDNA to limit of detection (LOD). **C,** A 72-year-old male treated by chemotherapy with radiologic PR after two TCs followed by SD after four TCs, but an increase in ctDNA, continuing throughout the follow-up period until radiologic PD. **D,** A 66-year-old male initially treated by combination treatment, experiencing radiologic PR and a consistent decrease in ctDNA, but an increase after shifting to maintenance treatment, while a radiologic unconfirmed PD was revealed, followed by PD. Thus, ctDNA could have clarified the nonconclusive radiologic evaluation. **E,** A 75-year-old male treated by nine TCs of immunotherapy before radiologically verified progression of the right adrenal gland, with a prior increase in ctDNA. Treatment by SBRT of the right adrenal gland, resulted in a concurrent decrease in ctDNA and radiologic SD, and the patient continued immunotherapy with no signs of PD. **F,** illustrates ctDNA measurements in long-term responders of immunotherapy; a significant drop in ctDNA to LOD, which persist. Gray dashed line illustrates one patient with suspected measurements of a subclonal mutation. LOD = Limit of detection. PD = Progressive disease. PR = Partial response. PD = Progressive disease. SD = Stable disease. SBRT = Stereotactic body radiotherapy. CTCAE = Common Criteria for Adverse Events. TC = Treatment Cycle.

In 73 of the 87 patients with longitudinally detectable ctDNA, radiologic PD was confirmed. Four patients died before radiologic PD, and 10 patients are long-term responders of immunotherapy without signs of radiologic PD, except for one patient experiencing PD 2 years after treatment initiation. Overall, molecular progression, defined as a ctDNA increase, was observed in 66 (90%) of 73 patients with PD—including a ctDNA increase before (*n* = 59) and immediately after radiologic PD (*n* = 7). In 7 patients (10%), no increase was observed. Examples of patient cases are illustrated in Fig. 4.

#### Lead Time

**FIGURE 5:**
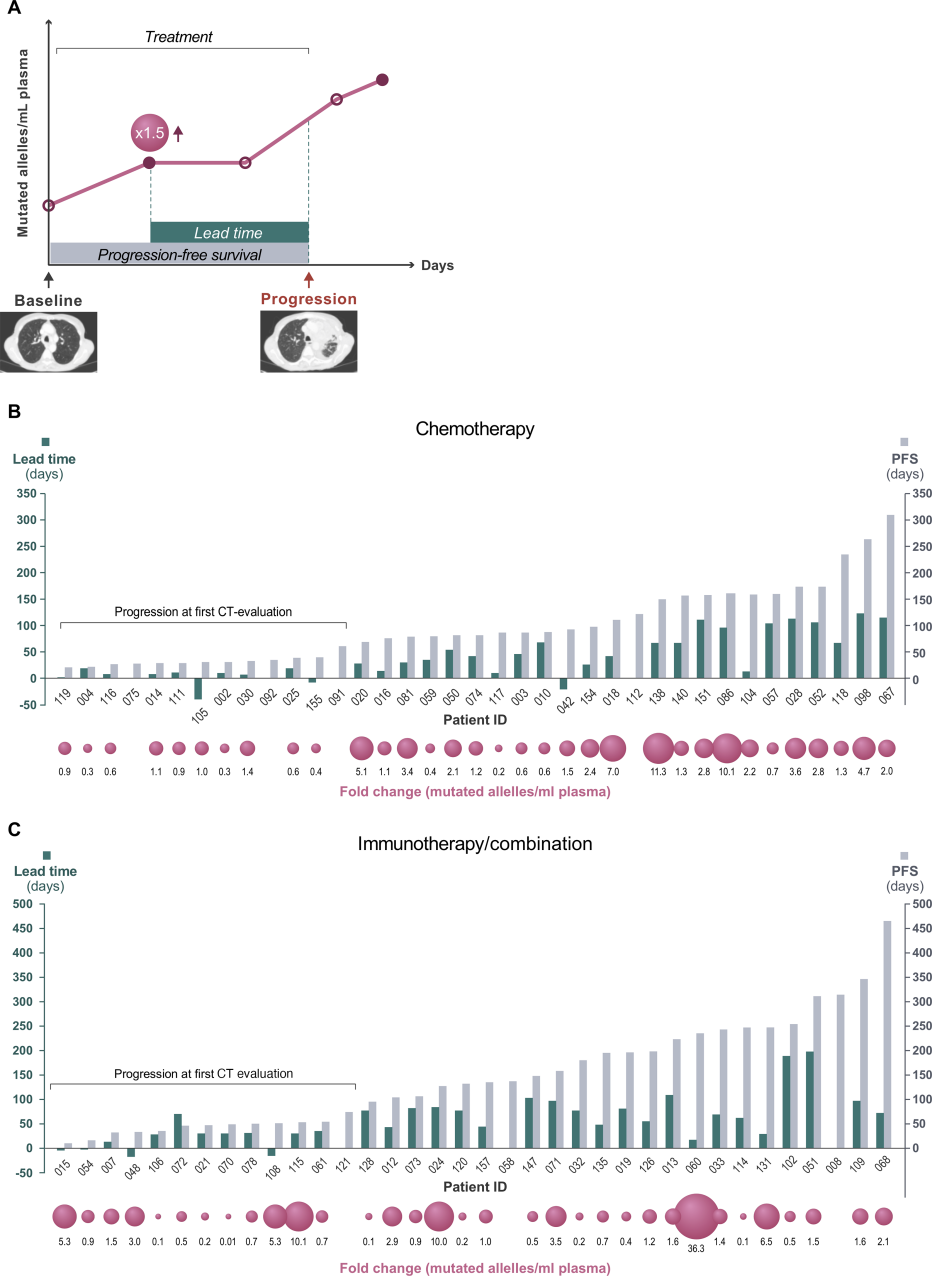
Lead time, PFS, and fold changes. **A,** A graphical illustration of lead time (green), fold change (pink ball), and PFS (gray). Lead time, PFS, and fold changes in patients treated by chemotherapy **B** and Immunotherapy/combination treatment **C**.

We noted the lead time between molecular progression and radiologic PD and the size of the increase of ctDNA (fold change) in each patient case including PFS—as illustrated in Fig. 5. Overall, we observed a median positive lead time of 51 days (range, 2–198) in patients with a ctDNA increase before radiologic PD (*n* = 59). A median negative lead time of 16 days (range, 3–40) was observed in patients with a ctDNA increase after radiologic PD (*n* = 7). In the immunotherapy/combination-treated patients and chemotherapy-treated patients a median positive lead time of 69 days (range, 13–198) and 42 days (range, 2–123), respectively, was observed.

#### Defining a Substantial Increase

To clarify whether a substantial or clinically relevant increase in ctDNA was present in each patient case, we used an approach by Thomsen and colleagues (41), defining a substantial increase as a ctDNA value with no overlap between the 95% CI of the current and immediately preceding measurements. In 28 (42%) of the 66 patients with a ctDNA increase before or proximately after progression, we found a ctDNA increase with no overlap between the 95% CI interval of the first increase and immediately preceding value. In addition, in 21 patients we observed an increasing ctDNA level after the first observed increase, which exceeded the 95% CI interval of the reference value, resulting in a total of 49 (74%) patients with a substantial increase.

As illustrated in Supplementary Fig. S5, a significant difference in both PFS (median 106 vs. 150 days; HR, 0.49; 95% CI, 0.31–0.77; *P* = 0.002) and OS (median 283 vs. 603 days; HR, 0.44; 95% CI, 0.27–0.72; *P* = 0.019) was observed in patients with a substantial increase in ctDNA (*n* = 49) versus patients without a substantial increase (*n* = 34), including long-term responders.

### Molecular Progression Correlated to Treatment Efficacy

**FIGURE 6:**
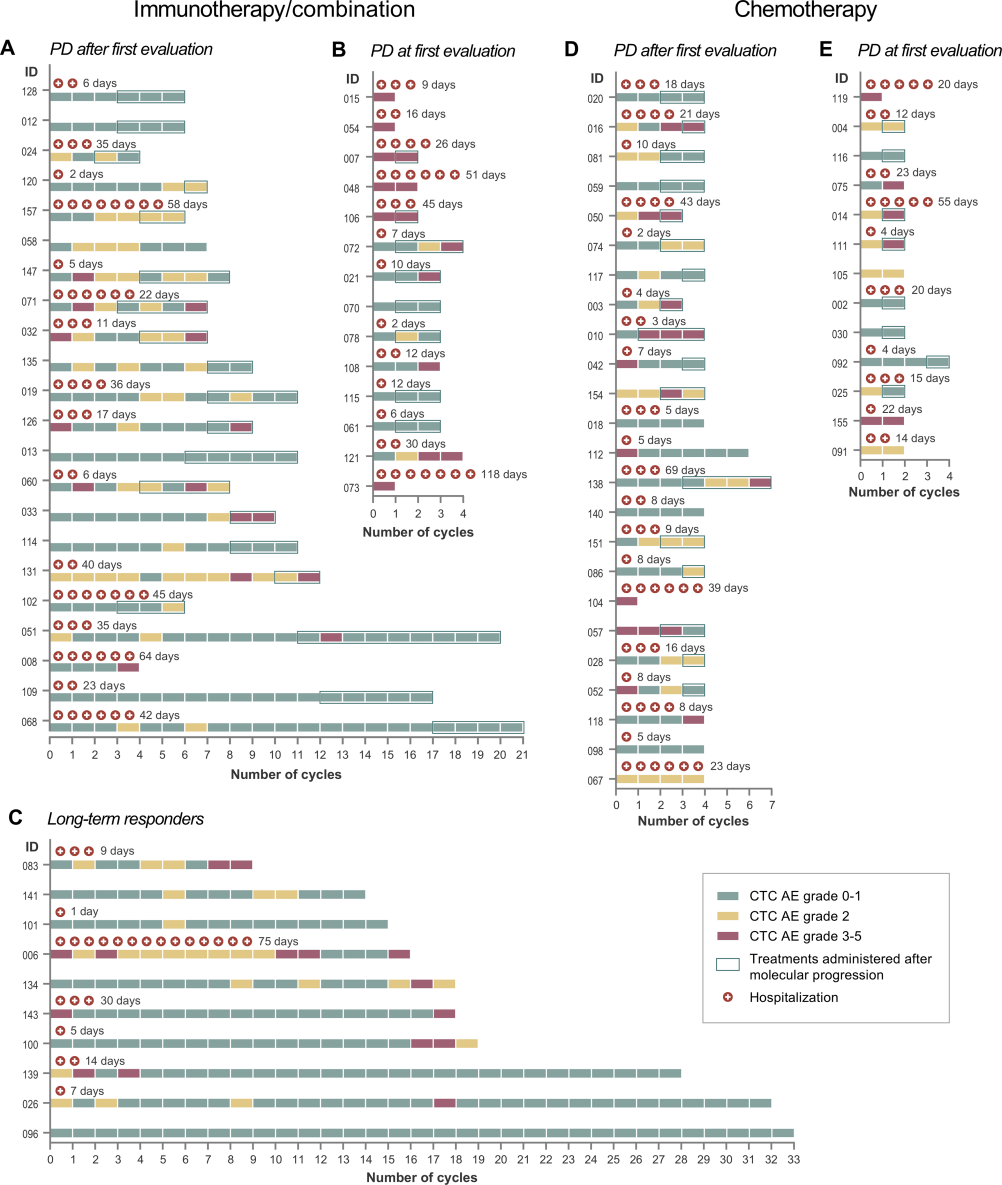
Treatment cycles administered before and after molecular progression, adverse events, number of hospitalizations, and total number of hospital admission days in each patient case. **A--C,** Immunotherapy/combination-treated patients. **D** and **E,** Chemotherapy-treated patients. CTCAE = Common Terminology Criteria for Adverse Events.

To investigate the potential clinical impact of molecular progression, we noted the cycles of treatments given before and after molecular progression, including a registration of adverse events, number and days of hospitalizations (Fig. 6). All patients with radiologically confirmed PD and longitudinally detectable ctDNA were included in the analyses (*n* = 73). In the chemotherapy-treated patient group (*n* = 37), a total of 37 (30%) of 123 treatment cycles were administered after molecular progression with a median of 1 cycle (range, 0–4). In the immunotherapy/combination-treated patient group (*n* = 36), a total of 82 (34%) of 242 treatment cycles were administered after molecular progression with a median of 2 cycles (range, 0–9). In 68% of chemotherapy-treated patients and 78% of immunotherapy/combination-treated patients treatment was administered after molecular progression. No obvious differences were observed in adverse events in treatments administered before and after molecular progression.

Approximately half of patients (52%, 58/111) experiencing PD received second-line treatment. The primary reason for not receiving second line treatment was a decline in performance status. Overall, a decline was observed in 58% (*n* = 64) of patients.

### Nonconclusive Radiologic Evaluations

Nonconclusive radiologic evaluations was defined by response evaluations that could not be categorized into complete response (CR), partial response (PR), PD, or SD according to the RECIST/iRECIST. Most common was dissociated response (mixed response) with responding and nonresponding lesions and pseudoprogression. In 48 (15%) of 330 evaluation scans performed, a nonconclusive result was noted. It was observed in 32 of 87 (37%) patients and most frequently in immunotherapy/combination-treated patients (*n* = 28), and less frequently in chemotherapy-treated patients (*n* = 4; Supplementary Fig. 6). We investigated whether ctDNA measurements immediately preceding or succeeding the nonconclusive evaluation scans could guide in the decision of treatment efficacy. In 38 (79%) of the 48 nonconclusive evaluation scans, we found correspondence between the proximate ctDNA measurement (increase/decrease) and the conclusion of the following evaluation scan. In majority of cases (*n* = 18), we observed a ctDNA decrease proximate to the nonconclusive evaluation scan, followed by SD, and less frequent we observed a ctDNA increase followed by PD (*n* = 14). In only few cases, we found a ctDNA decrease, followed by PR (*n* = 5), and CR (*n* = 1). In eight cases of nonconclusive scans, no correspondence between the proximate ctDNA measurements and the following scan evaluations was observed, illustrated by a decrease before PD (*n* = 5) and a ctDNA increase before SD (*n* = 3). In 1 patient with two nonconclusive evaluation scans, the proximate ctDNA measurements were nonconclusive. Patient examples are illustrated in Fig. 4B and D. Details of treatment administration, results of evaluation scans and ctDNA dynamics in each patient case are illustrated in Supplementary Fig. S7.

## Discussion

The clinical relevance and strategy for ctDNA treatment monitoring in advanced NSCLC is currently being defined. Explorative, retrospective studies including highly selected patients with limited numbers of blood samples and different analytic methodologies have been the foundation of our current knowledge. Real-life studies are required to explore clinical validity, paving the way for the needed interventional studies of clinical utility.

### Detection of ctDNA by ddPCR

Measurements of ctDNA by ddPCR has a favorable high analytic sensitivity and specificity (42, 43), but are limited by the number of mutational assays available, when performing analyses in cohorts of patients with different driver mutations. Thus, many liquid biopsy studies in advanced NSCLC have focused on selected patients with prespecified driver mutations (10, 14, 24, 44). By designing mutation-specific assays in patient cases without a commercially available assay, we succeeded in detecting ctDNA in 67% of patients in our real-life cohort (Fig. 3D). To our knowledge, reporting of ddPCR analyses performed in a similar, real-life cohort of patients with advanced NSCLC without targetable mutations, have been missing.

Application of a multi-gene approach by either multiplex ddPCR or next-generation sequencing (NGS) is gaining increasing interest in the research field of ctDNA in advanced NSCLC (9, 13, 16–20, 22, 23, 27, 28), offering advantages in exploring a wide range of molecular alterations. Recently, Zhang and colleagues (23), reported on ctDNA measurements in one of the largest cohorts of patients with 16 different tumor types (*n* = 978), receiving immunotherapy, using a 73 gene panel (Guardant360).

Despite decreasing cost of NGS-based methods, a clear economic advantage is still present by the ddPCR methods. In addition, a short turnaround time makes it clinically feasible.

### ctDNA Measurements Correlate to PFS/OS

Previous studies have, across different methodologies, thresholds, and methods of reporting; all demonstrated a clear correlation between baseline ctDNA measurements and/or early ctDNA dynamics and treatment efficacy (8–23) and OS (8, 9, 12, 14–19, 22, 23). Our study confirmed these findings by illustrating a correlation between baseline ctDNA measurements below median, or nondetectable ctDNA, and a longer OS (Supplementary Figs. S2 and S3).

From a clinical perspective, it is interesting to have the knowledge of predicted PFS and OS from baseline ctDNA measurements, but it will not change decisions of first-line treatment if the patient has an acceptable PS to receive systemic treatment. Contrarily, a certainty of how to interpret single ctDNA measurements during treatment can have great clinical impact on whether to continue or discontinue a treatment.

### Early Detection of Treatment Failure

Clinical deterioration is one of the greater challenges in treating patients with advanced NSCLC (4, 5). Symptoms of progression and adverse events are the main reasons for a decline in PS, which was observed in almost 60% of patients in our study cohort at time of PD with only half of patients capable of receiving second-line treatment. Thus, a precise and reliable method of evaluating treatment efficacy is essential to reduce long-lasting ineffective treatments and to spare patients from needless adverse events.

By longitudinal measurements of ctDNA, we observed an increase in ctDNA (= molecular progression) in majority (90%) of patients with radiologic PD, indicating a correlation between molecular progression and radiologic PD. Former studies have demonstrated similar results; however, primarily focusing on early response assessment in immunotherapy-treated cohorts, including only few longitudinal ctDNA measurements (10–18, 20, 21). A potential clinically relevant observation was that the molecular progression was observed 51 days (range, 2–198) before radiologic PD (Fig. 5), similar to the findings by Goldberg and colleagues (28). From a biological perspective, it could be explained by the ability to detect even small molecular changes, indicating progression at a very early timepoint, before radiologic evidence of PD—see examples in Fig. 4A–C. The lead time was even more pronounced (∼2 months) in patients treated by immunotherapy/combination treatment, which is possibly correlated to the highest frequency of nonconclusive radiologic evaluations. Of note, these findings only represent the subgroup of patients with detectable ctDNA and a ctDNA increase before radiologic PD (81%).

### The Possibility to Discontinue Inefficient Treatment

By defining molecular progression as a proxy of true progression and converting it to a clinically relevant measurement, we could potentially reduce the number of administered treatment cycles by approximately 30% (Fig. 6). Around 75% of patients received one or more cycles of likely futile treatment. According to Spear and colleagues, the treatment response rates in oncology is of only 25% (45), thus underlining the necessity of a reliable method of treatment monitoring. To our knowledge, no prior studies have reported on the clinical impact of early detection of treatment failure in terms of number of potentially spared treatment cycles. Of importance, these findings need interpretation with precaution and are not directly generalizable to the clinical setting as important clinical factors need to be included in treatment decision-making, like subjective symptoms, objective measurements, treatment options, and patients’ perspectives.

Defining true progression is important, particularly in advanced NSCLC, covering a limited numbers of treatment lines. Yet, a clear definition of a substantial ctDNA increase has not been clarified and studies have different methods of reporting including different definitions (29). By comparing 95% CI intervals of subsequent measurements, we found a substantial increase in 74% of patients. Of note, we measured ctDNA until confirmed radiologic PD and thus expected all patients to have a substantial increase at some timepoint during treatment or follow-up—except for long-term responders. Intriguingly, we observed a significant difference in PFS and OS in patients with/without a substantial increase in ctDNA (Supplementary Fig. 5).

### Long-term Responders

Long-term responders are defined as the “tail” of the PFS/OS curve in immunotherapy-treated patients, but a clear definition of PFS in long-term responders has not yet been clarified (46, 47). In our cohort, we observed 10 (21%) of 47 immunotherapy/combination-treated patients, without PD until the cut-off date of March 1, 2022, except for one patient with PD after 622 days. All patients exhibited a certain response in the ctDNA dynamics; a consistent drop in ctDNA to a level below limit of detection in the beginning of the treatment course (<3.5 months), which persisted (Fig. 4F). These findings suggest that early ctDNA dynamics are predictive of benefit from immunotherapy, which corresponds to findings by other studies (10–18, 20, 21, 23).

### Can ctDNA Measurements Clarify Radiologic Evaluations?

Nonconclusive radiologic evaluations are a major challenge in the treatment of patients with advanced NSCLC, and the frequency has increased after implementation of immunotherapy in the treatment guidelines, due to both dissociated response (48) and pseudoprogression—the latter observed in 5%–10% of patients treated by immune checkpoint inhibitors (49, 50). Beside the objective and subjective clinical evaluation, no other measurements can support the clinician's decision of whether to continue or discontinue an ongoing treatment. We observed nonconclusive radiologic evaluations, including cases of pseudoprogression in 15% of all radiologic evaluations in our study, corresponding to 37% of patients—and most frequently observed in immunotherapy/combination-treated patients (see Supplementary Fig. S6; Fig. 4B and D). In 79% of the nonconclusive radiologic evaluations, we found that the dynamics of the proximate ctDNA measurements (increase/decrease) reflected the results of the following evaluation scans with the potential of detecting both SD and PD earlier. These results are in line with results of a study of patients with immune checkpoint–treated melanoma, concluding that ctDNA profiles can differentiate pseudoprogression from true progression (50). In addition to these findings, Zhang and colleagues (23) found that ctDNA dynamics can help in early differentiation of responders among patients with radiologically SD at first evaluation.

### Limitations

Our study has some limitations that need to be addressed and included in the interpretation of the results. The use of a single-mutation approach in detection of ctDNA has the disadvantage of potentially missing ctDNA dynamics and/or clonality of other, and possibly more important mutations, including the risk of tracking subclonal mutations.

We did not systematically perform analyses to account for CHIP and germline variants, for example*,* by sequencing of whole blood—except for one case, as earlier described—although we did measure potential white blood cell DNA contamination and performed quality control. The hard cutoff of ≥3 positive droplets could potentially influence the proportion of patients with nondetectable ctDNA due to false-negative results. In addition, analyses by noise-informed calling methods could improve ctDNA detection by lowering the false-positive rate (51).

We reported on patients with detectable ctDNA (67%) and consequently our conclusions can be affected by selection bias. In addition, in 8 patients, no cancer-specific mutations were found by the TSO500 HT gene panel (Fig. 2), questioning whether broader genomic profiling/exome sequencing should be preferred in the clinical setting.

Subgrouping of patients based on treatment regimens reduces the number of patients in each group and thus consequently limits the conclusions that can be drawn.

Another aspect to be addressed was the calculation of lead time from molecular progression to radiologic progression. As we accepted one scan showing stable disease in-between molecular and radiologic progression, it could be a false-positive molecular progression. We chose to trust the ctDNA measurements and interpreted the results as an earlier precursor of disease progression. To more precisely investigate the correlation between ctDNA measurements and the results of evaluation scans, simultaneous blood sampling and scan evaluation could have been performed to reduce lead time bias and in order to calculate positive and negative predictive values.

### Perspectives

Are we ready for interventional studies of ctDNA in advanced NSCLC to clarify the use of ctDNA measurements in clinical practice? On the basis of our “real-life” study and important recent larger “proof-of-concept” studies, a correlation between ctDNA dynamics and treatment efficacy has been demonstrated. Yet, the optimal strategy for ctDNA measurements have not been defined, which includes optimizing methods of detection and defining and validating thresholds for ctDNA increases/decreases as surrogate markers of treatment response (ctDNA RECIST; ref. 29). Further analytic and clinical validation is needed, before moving to the next step of investigating clinical utility (52). Newly published European Society for Medical Oncology (ESMO) recommendations on the use of ctDNA assays with cancer (2022), recommends randomized, interventional studies before clinical utility of ctDNA measurements in treatment monitoring of advanced cancers (53).

A proposed set-up of an interventional study could be treatment monitoring of patients with advanced NSCLC, receiving immunotherapy with plasma-based measurements of ctDNA before every treatment cycle and at time of evaluation scans. Immunotherapy-treated patients would have great advantages of ctDNA monitoring by an early distinguishing between nonresponders and long-term responders. In addition, ctDNA measurements could potentially clarify the frequent nonconclusive radiologic evaluations. The methodology could either be NGS or multiplex ddPCR, preceded by broad genomic profiling of tissue or plasma. Different advantages and disadvantages are related to either a tumor-agnostic or tumor-informed strategy and the optimal strategy has not yet been defined (53). A recent published study with pooled data from five different immunotherapy-treated NSCLC cohorts (*n* = 365), proposed a predictive model of durable response, consisting of seven of the most predictive genes (54).

A potential limitation in the clinical implementation of ddPCR-based treatment monitoring is the need to design and validate patient-specific assays within a short time span, which requires resources and expertise. A national or international database with information on validated assays could accelerate and improve the clinical use.

In a proposed interventional study, a randomization between ctDNA-based monitoring and radiologic monitoring should be performed at baseline. Discontinuation of treatment should be based on an increase in ctDNA and stable/decreasing ctDNA in continuation of treatment. A confirmatory ctDNA measurement could be considered in case of an increase to account for the uncertainties of single ctDNA measurements. Furthermore, we would propose discontinuation of immunotherapy before the standard 2 years of treatment, if ctDNA measurements reach the limit of detection, based on the certain ctDNA response we observed in all long-term responders. Thereby, it would be possible to explore the optimal duration of immunotherapy, as empasized in recent studies (55–57). A noninferiority study design is required with primary endpoint being OS—and secondary endpoints being PFS by radiologic evaluation and by ctDNA measurements, number of treatment cycles, adverse events, and quality of life.

## Conclusion

Our study has contributed with additional knowledge on long-term measurements of ctDNA in a real-life cohort, linking results to a clinical context. In 90% of patients experiencing PD, we observed an increase in ctDNA, defining molecular progression. It was observed 1.5 months before verified radiologic progression and could potentially spare patients from 30% of likely inefficient treatment cycles. In addition, ctDNA measurements could clarify the results of nonconclusive radiologic evaluations in the vast majority of cases.

## Supplementary Material

Supplementary Figure FS1 OS and PFS

Supplementary Figure FS2 OS and PFS – detectable/non-detectable

Supplementary Figure FS3 OS and PFS – above/below median

Supplementary Figure FS4 OS and PFS – cfDNA

Supplementary Figure FS5 OS and PFS – substantial/no substantial increase

Supplementary Figure FS6 Patients with non-conclusive scan evaluations

Supplementary Figure FS7 All patient cases

Supplementary Table TS1 Sequencing data of tumor tissue in each patient case

Supplementary Table TS2 Primer sequences

Supplementary Table TS3 Representativeness of study participants

Supplementary Table TS4 Analyses of variables for ctDNA detection
