# Supplementary figures and images for: Circulating Tumor DNA Monitoring Reveals Molecular Progression before Radiologic Progression in a Real-life Cohort of Patients with Advanced Non–small Cell Lung Cancer
Source: Cancer Res Commun. 2022 Oct 13;2(10):1174–87. doi: 10.1158/2767-9764.CRC-22-0258 (PMC10035379; doi:10.1158/2767-9764.CRC-22-0258)

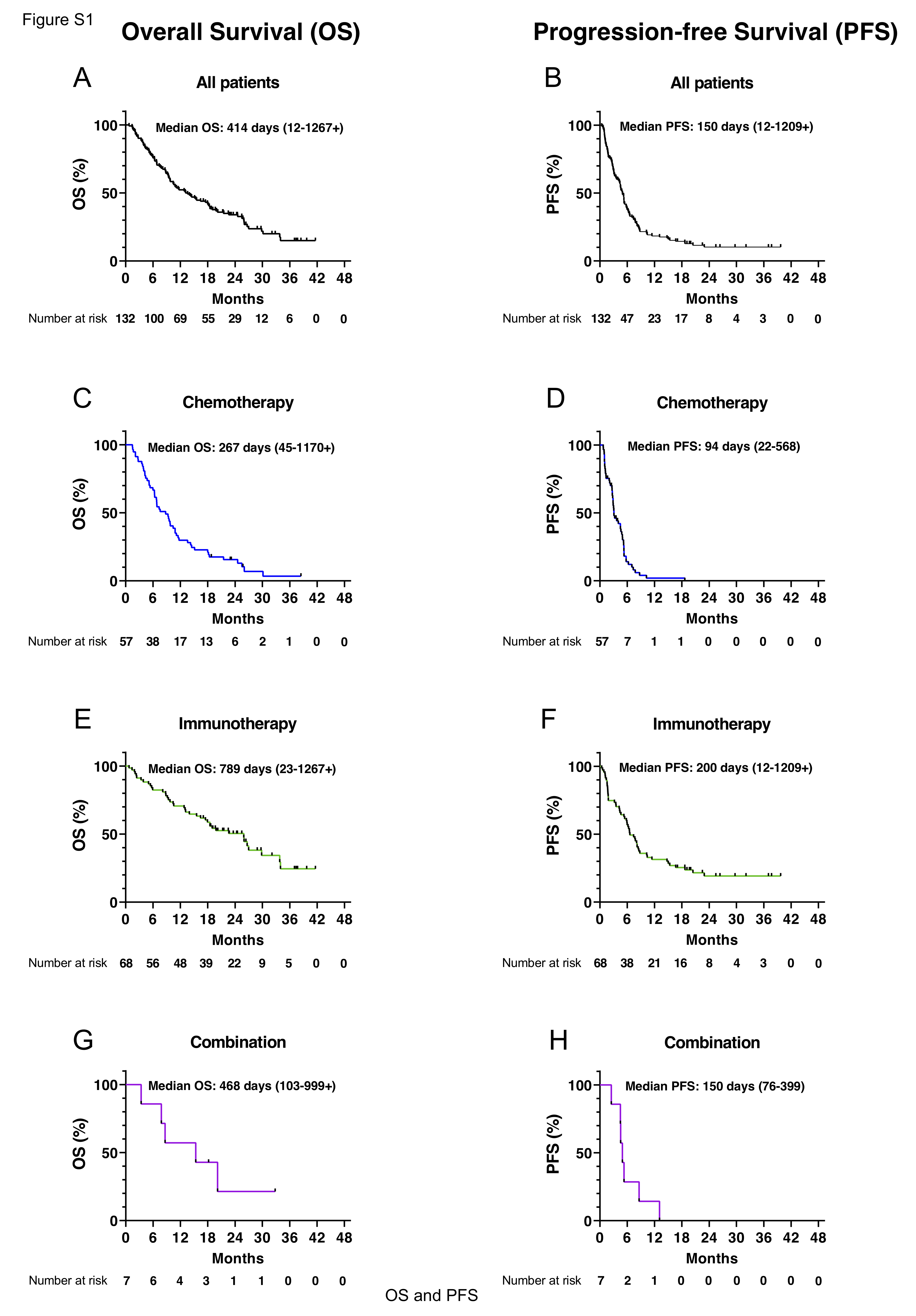

Supplement: Supplementary Figure FS1 — OS and PFS [file crc-22-0258-s01.png]

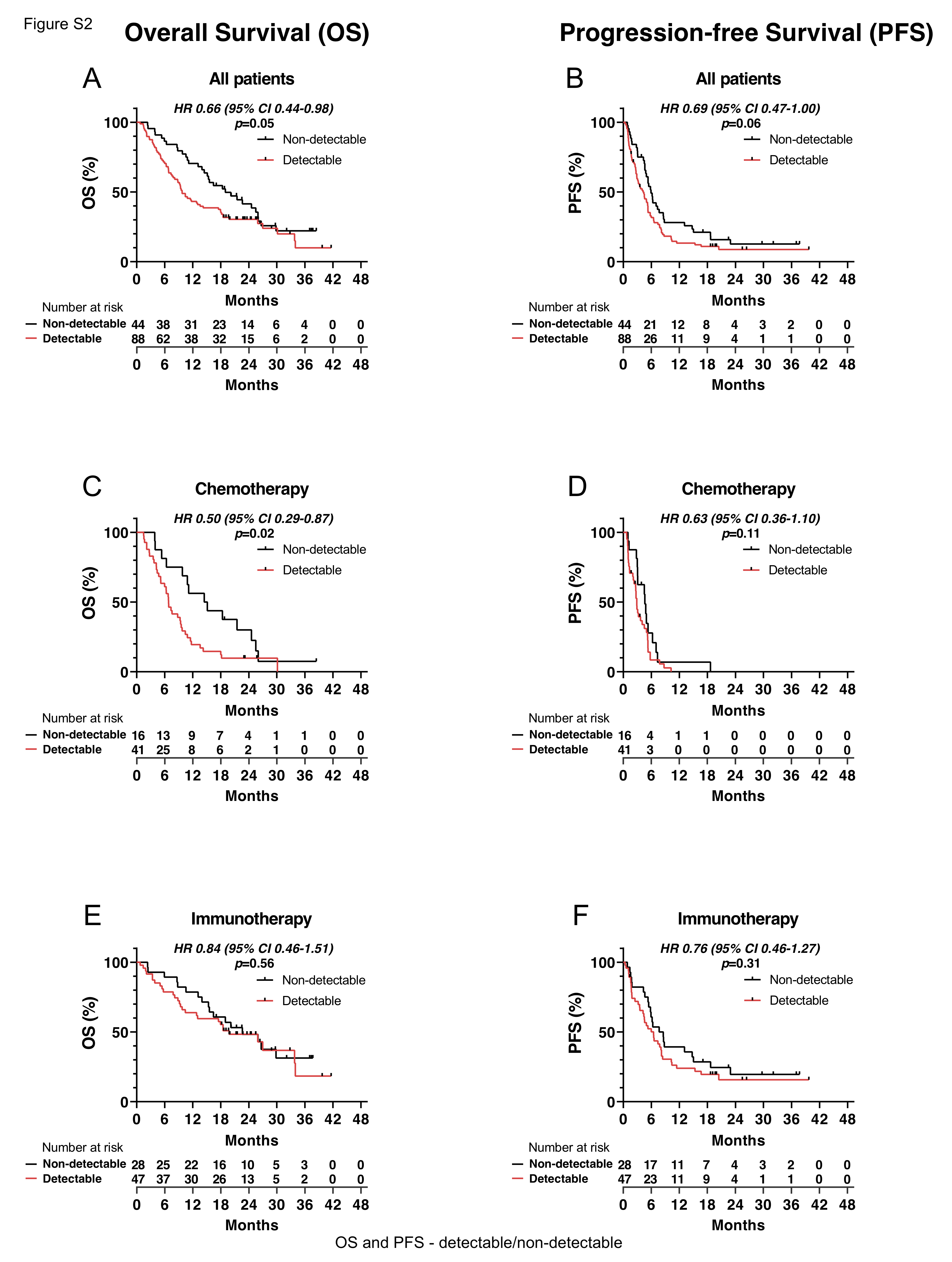

Supplement: Supplementary Figure FS2 — OS and PFS – detectable/non-detectable [file crc-22-0258-s02.png]

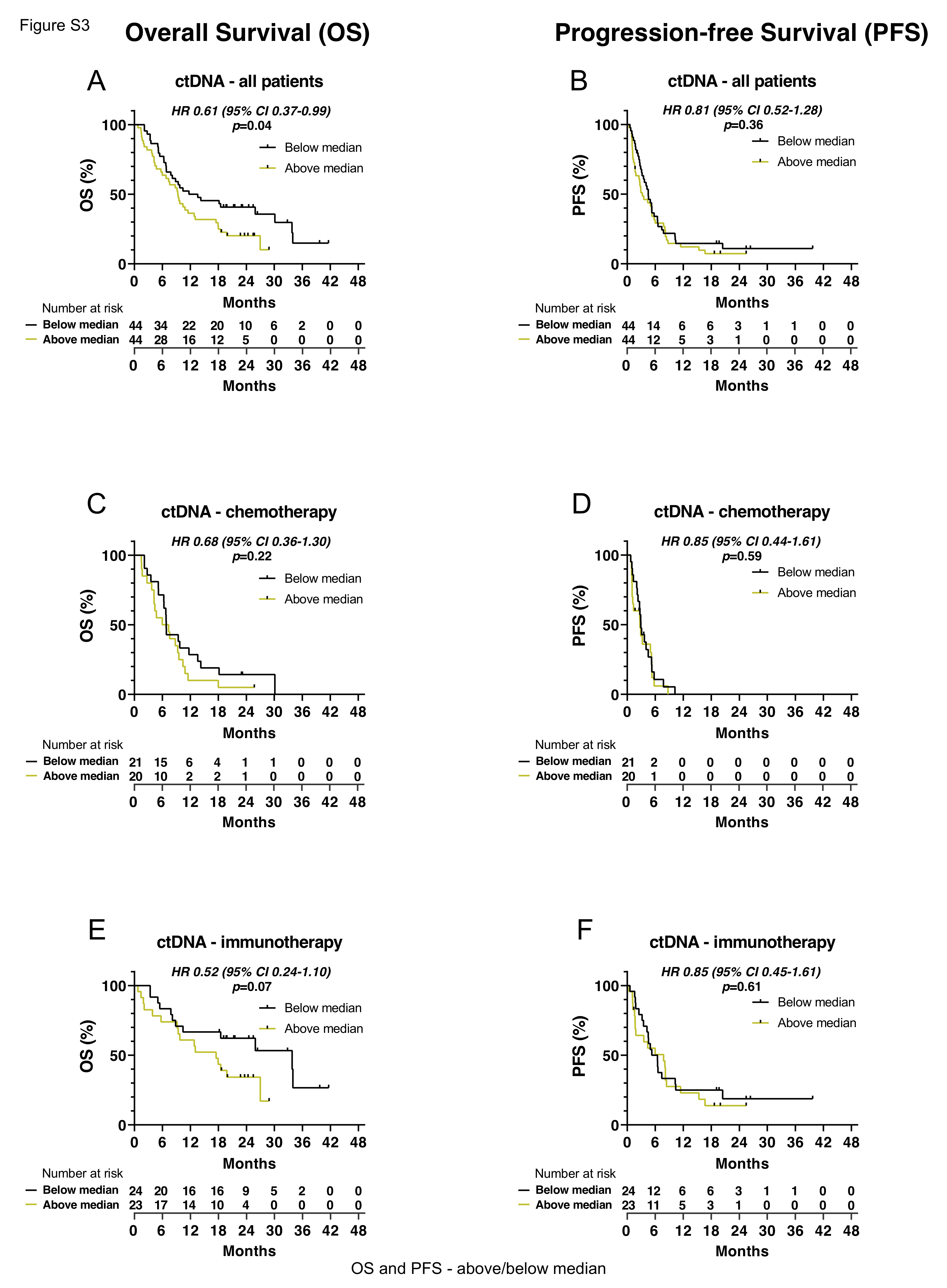

Supplement: Supplementary Figure FS3 — OS and PFS – above/below median [file crc-22-0258-s03.png]

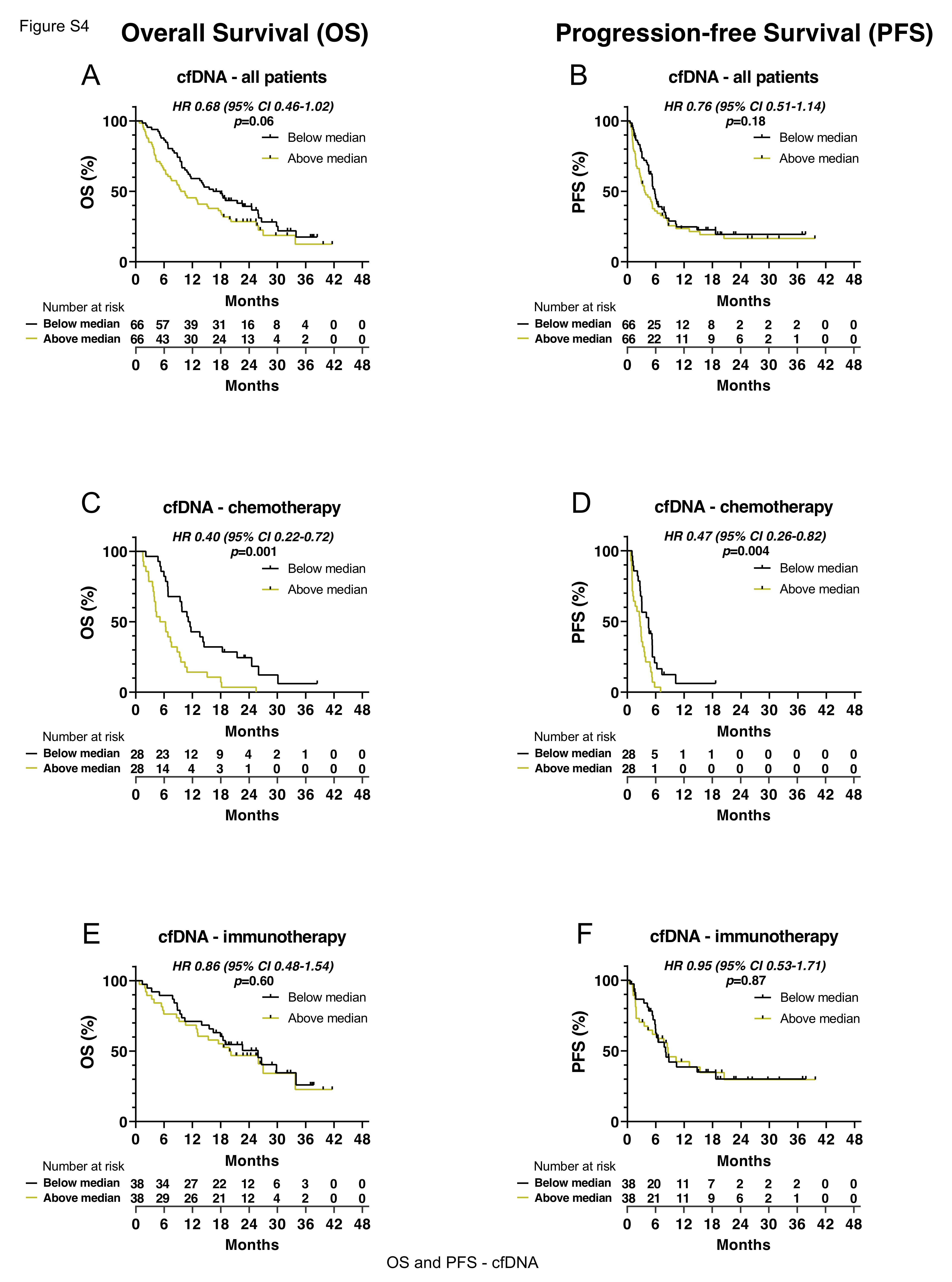

Supplement: Supplementary Figure FS4 — OS and PFS – cfDNA [file crc-22-0258-s04.png]

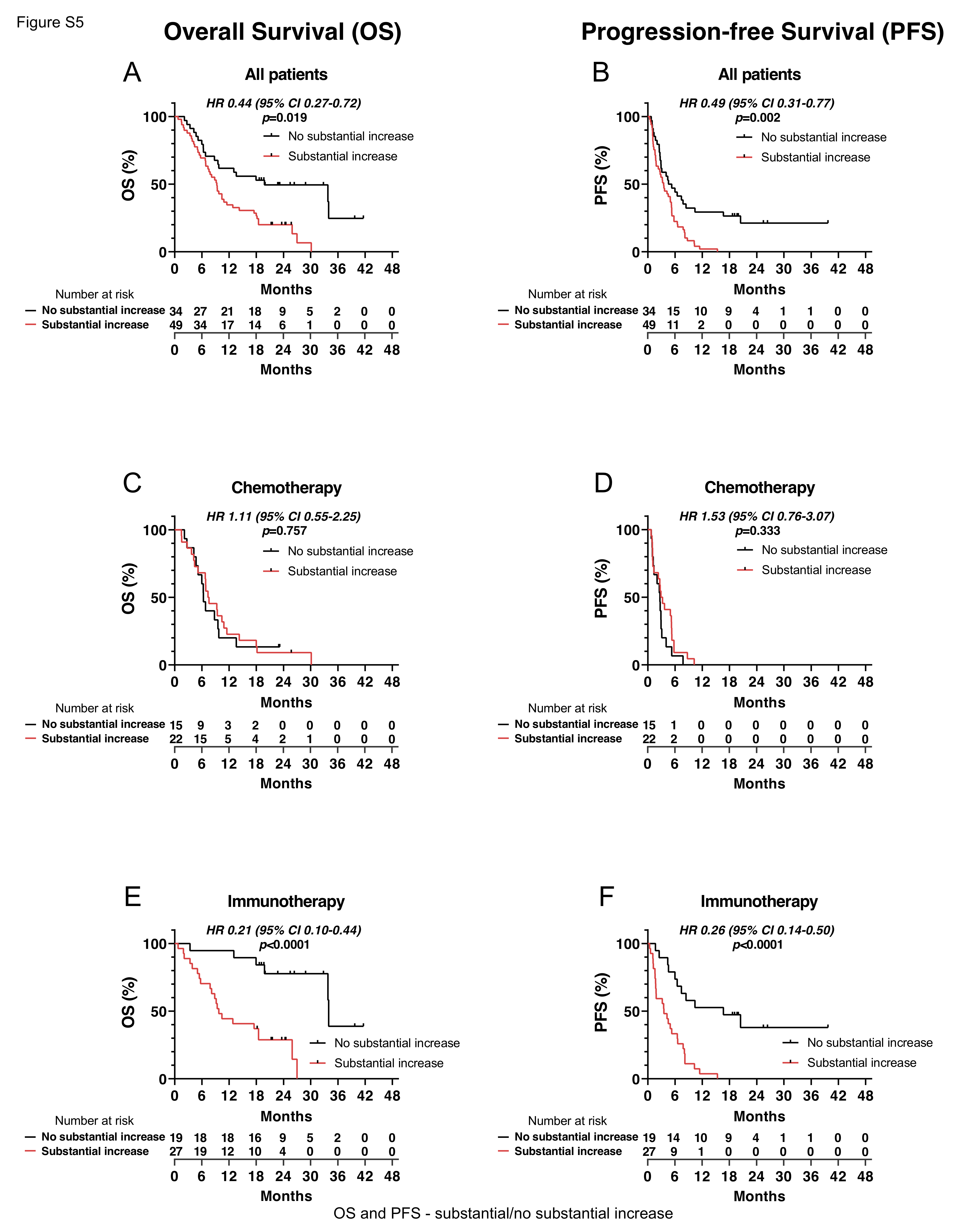

Supplement: Supplementary Figure FS5 — OS and PFS – substantial/no substantial increase [file crc-22-0258-s05.png]

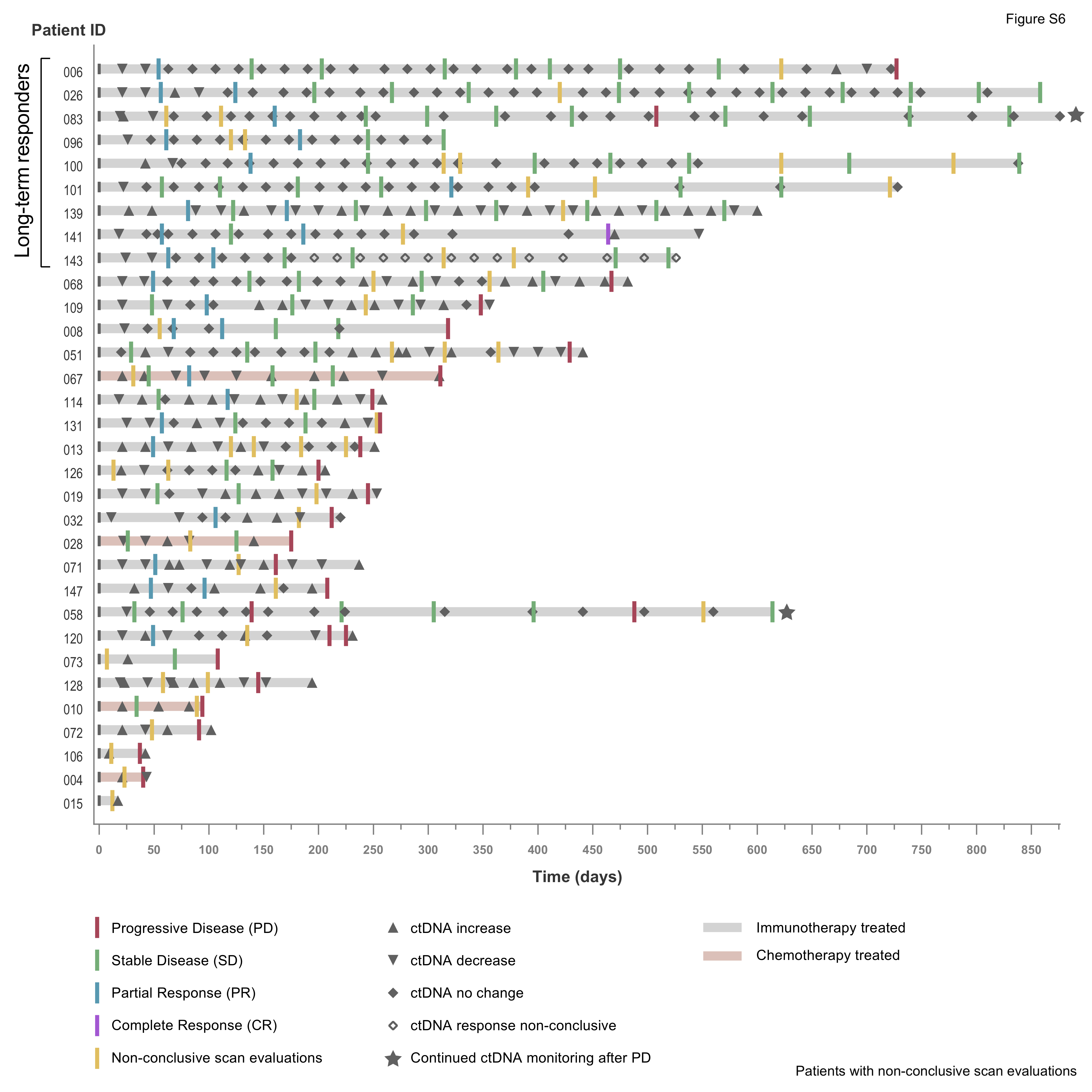

Supplement: Supplementary Figure FS6 — Patients with non-conclusive scan evaluations [file crc-22-0258-s06.png]
