# Supplementary material for: Circulating Tumor DNA Monitoring Reveals Molecular Progression before Radiologic Progression in a Real-life Cohort of Patients with Advanced Non–small Cell Lung Cancer
Source: Cancer Res Commun. 2022 Oct 13;2(10):1174–87. doi: 10.1158/2767-9764.CRC-22-0258 (PMC10035379; doi:10.1158/2767-9764.CRC-22-0258)
Supplement: Supplementary Figure FS7 — All patient cases [file crc-22-0258-s07.pdf]

Figure S7

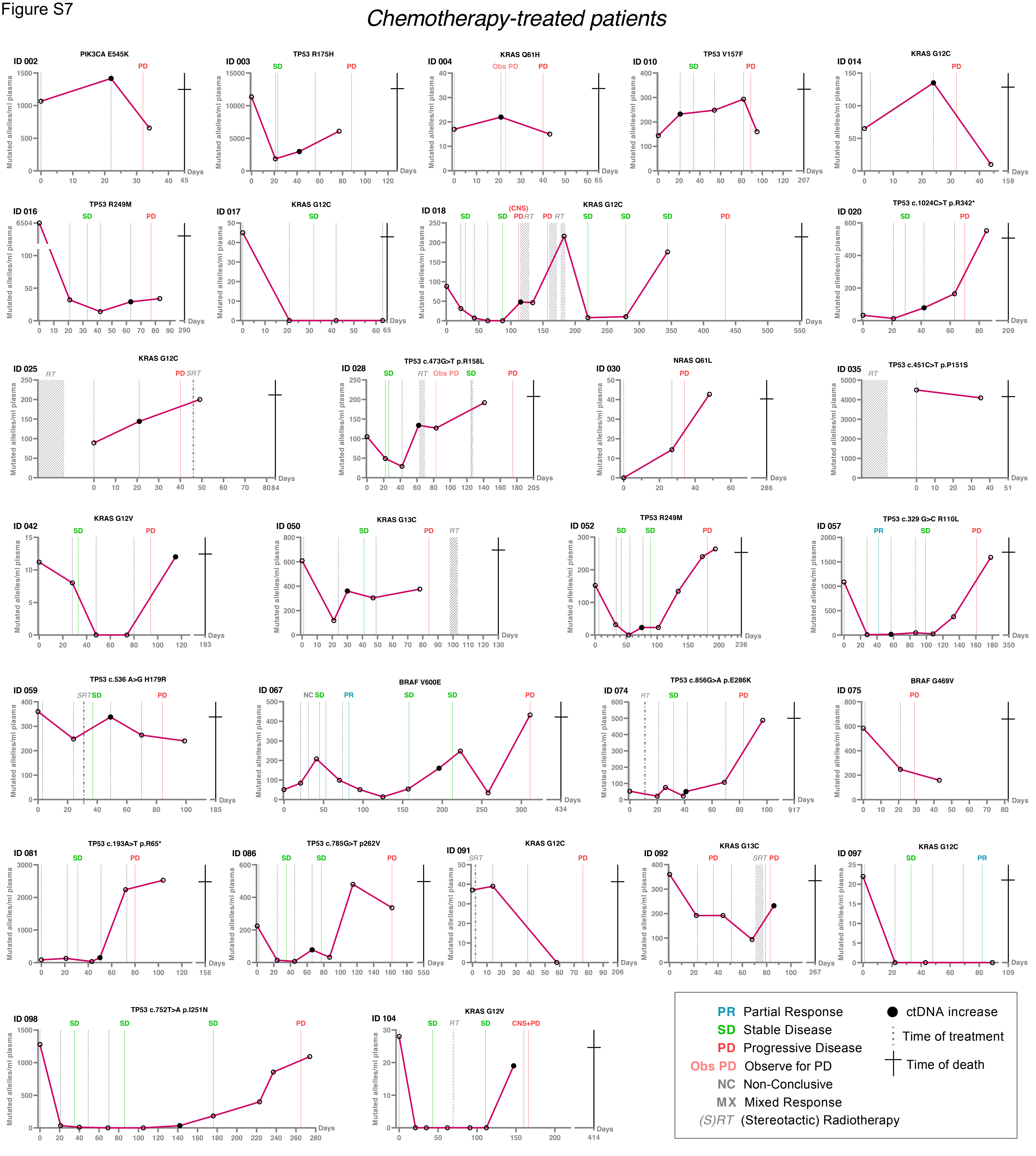
Chemotherapy-treated patients
ID 002
PIK3CA E545K
ID 003
TP53 R175H
ID 004
KRAS Q61H
ID 010
TP53 V157F
ID 014
KRAS G12C
ID 016
TP53 R249M
ID 017
KRAS G12C
ID 018
KRAS G12C
(CNS)
ID 020
TP53 c.1024C>T p.R342*
ID 025
KRAS G12C
ID 028
TP53 c.473G>T p.R158L
ID 030
NRAS Q61L
ID 035
TP53 c.451C>T p.P151S
ID 042
KRAS G12V
ID 050
KRAS G13C
ID 052
TP53 R249M
ID 057
TP53 c.329 G>C R110L
ID 059
TP53 c.536 A>G H179R
ID 067
BRAF V600E
ID 074
TP53 c.856G>A p.E286K
ID 075
BRAF G469V
ID 081
TP53 c.193A>T p.R65*
ID 086
TP53 c.785G>T p262V
ID 091
KRAS G12C
ID 092
KRAS G13C
ID 097
KRAS G12C
ID 098
TP53 c.752T>A p.I251N
ID 104
KRAS G12V
CNS+PD
Mutated allelles/ml plasma
Days
PR Partial Response
SD Stable Disease
PD Progressive Disease
Obs PD Observe for PD
NC Non-Conclusive
MX Mixed Response
(S)RT (Stereotactic) Radiotherapy
ctDNA increase
Time of treatment
Time of death

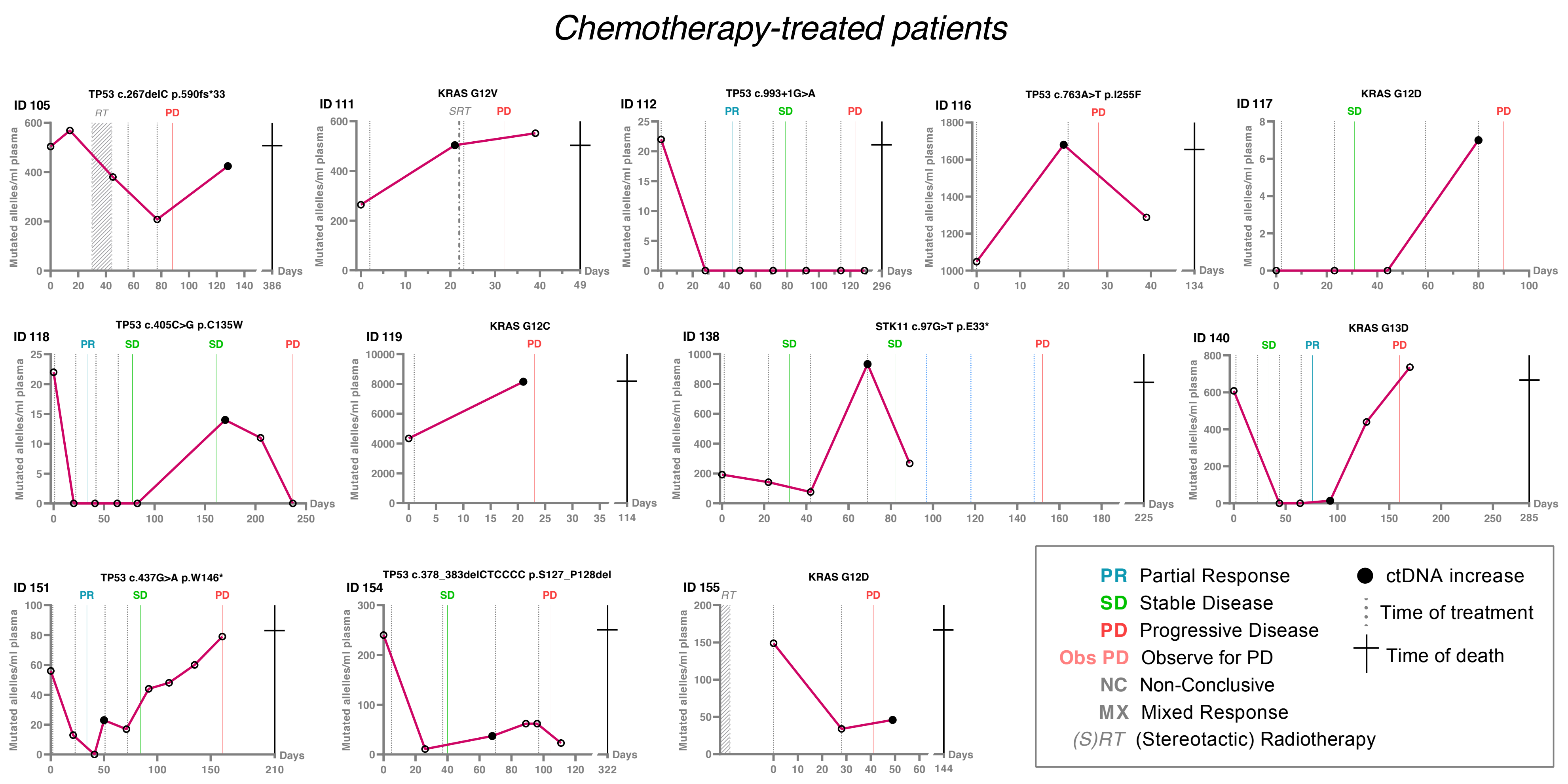

Chemotherapy-treated patients
ID 105
TP53 c.267delC p.590fs*33
ID 111
KRAS G12V
ID 112
TP53 c.993+1G>A
ID 116
TP53 c.763A>T p.I255F
ID 117
KRAS G12D
ID 118
TP53 c.405C>G p.C135W
ID 119
KRAS G12C
ID 138
STK11 c.97G>T p.E33*
ID 140
KRAS G13D
ID 151
TP53 c.437G>A p.W146*
ID 154
TP53 c.378_383delCTCCCC p.S127_P128del
ID 155
KRAS G12D
Mutated alleles/ml plasma
Days
PR Partial Response
SD Stable Disease
PD Progressive Disease
Obs PD Observe for PD
NC Non-Conclusive
MX Mixed Response
(S)RT (Stereotactic) Radiotherapy
ctDNA increase
Time of treatment
Time of death

Immunotherapy-treated patients

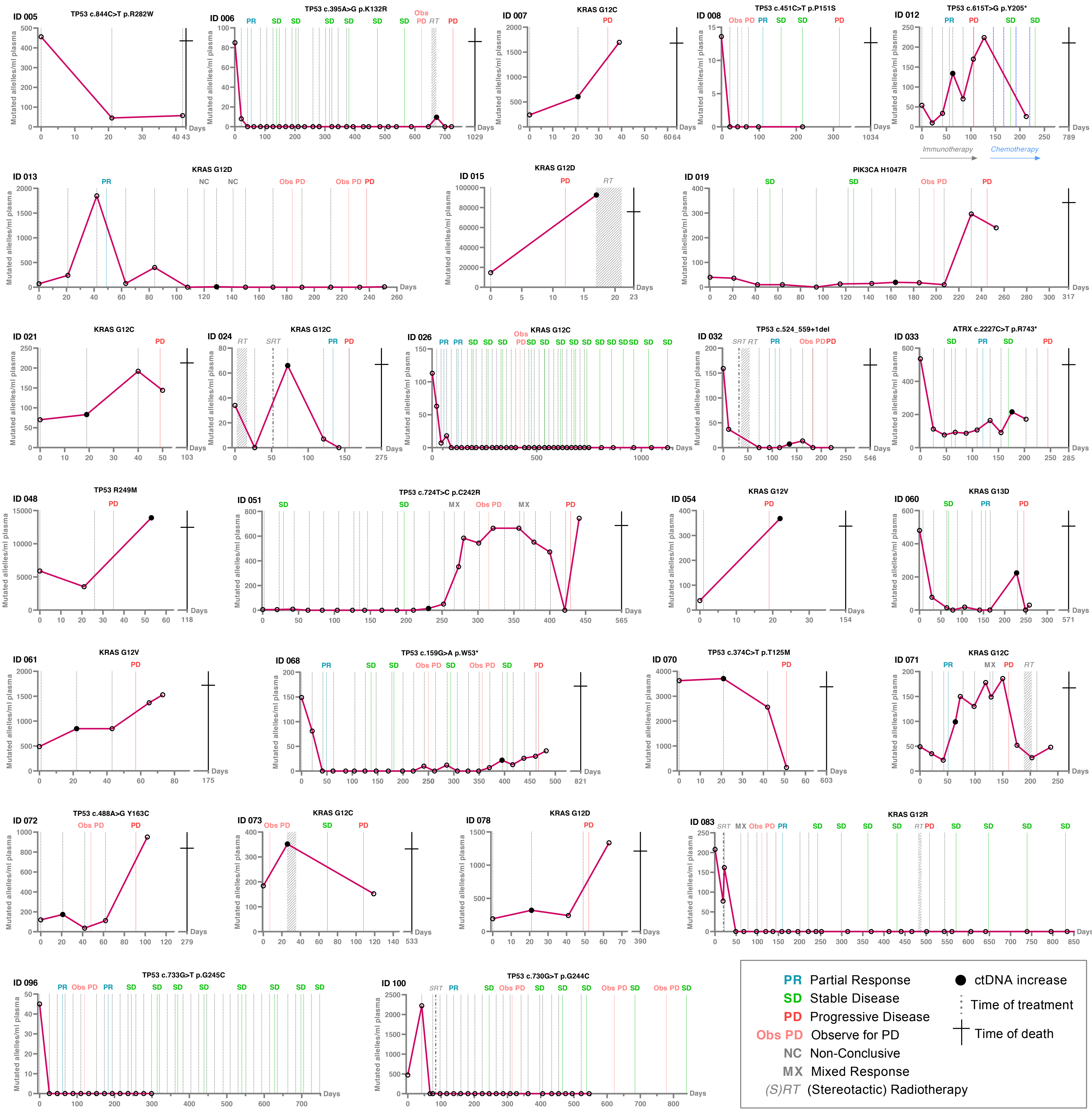

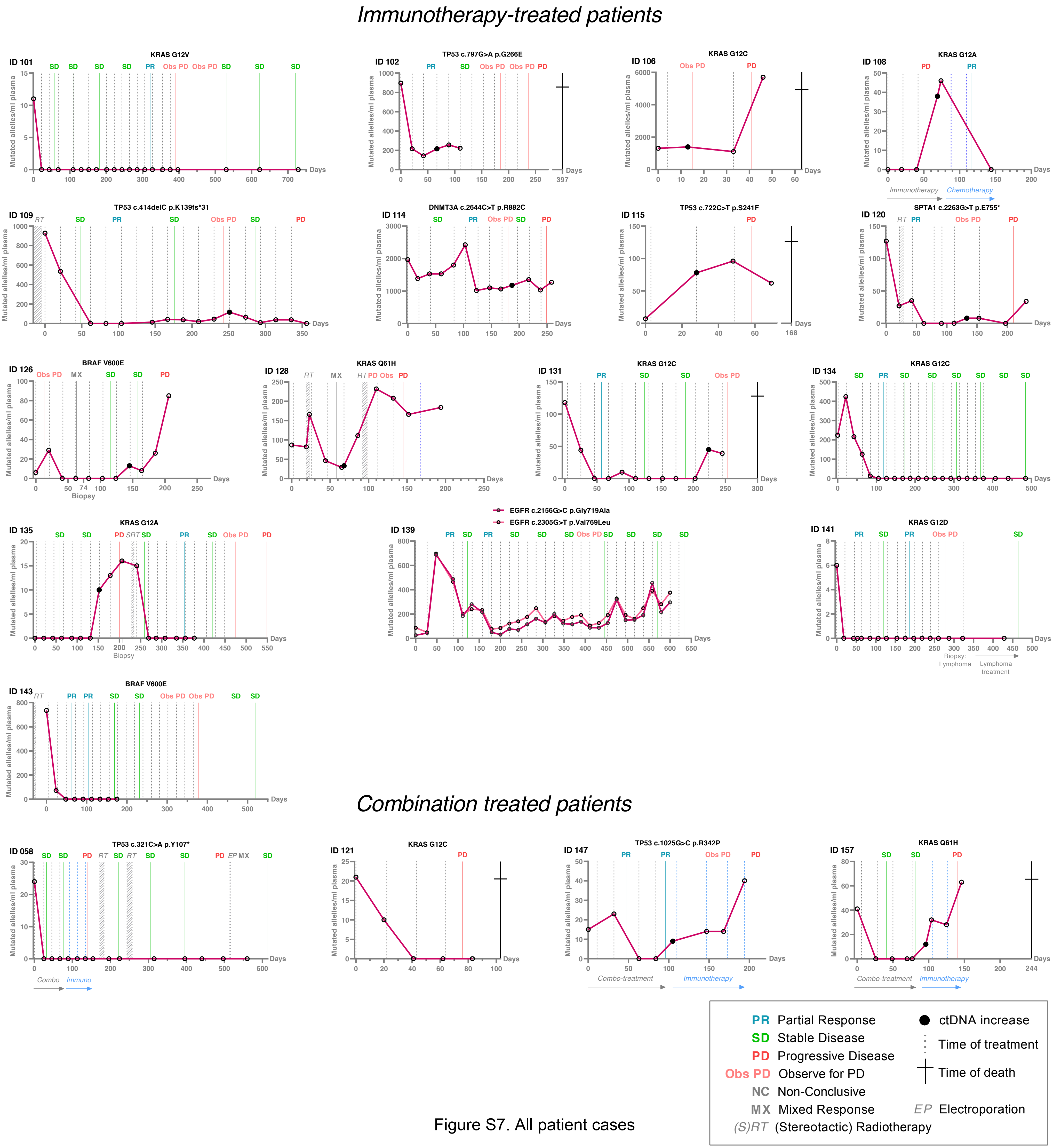

Figure S7. All patient cases
