# Supplementary material for: Circulating Tumor DNA Monitoring Reveals Molecular Progression before Radiologic Progression in a Real-life Cohort of Patients with Advanced Non–small Cell Lung Cancer
Source: Cancer Res Commun. 2022 Oct 13;2(10):1174–87. doi: 10.1158/2767-9764.CRC-22-0258 (PMC10035379; doi:10.1158/2767-9764.CRC-22-0258)
Supplement: Supplementary Table TS3 — Representativeness of study participants [file crc-22-0258-s10.pdf]

Table S3

Supplementary Table 3. Representativeness of Study Participants	
Cancer type /subtype	Non-Small Cell Lung Cancer (NSCLC)
Stage	Advanced/non-curative locally advanced
Considerations related to:	
Sex	The incidence of NSCLC is almost equally distributed among men and women, which was reflected in our study cohort.
Age	The median age of people diagnosed with lung cancer (all stages) in US and Europe is approximately 70 years. In our cohort, only patients receiving systemic treatment were included, resulting in a median age of 66 years (range: 44-84).
Smoking status	Smoking is the leading risk factor for lung cancer, accounting for approximately 80-90% of lung cancer cases. In our study cohort 85% were current or former smokers.
Histology	Adenocarcinoma histology is the most common subtype of NSCLC, accounting for 40-50%. In our study cohort a larger proportion of patient had adenocarcinoma histology (72%).
Programmed Death-Ligand1 (PD-L1) tumor proportion score	PD-L1 tumor proportion score (TPS) is a biomarker of response to immune-checkpoint inhibitors in advanced NSCLC with approximately 30% having a PD-L1 TPS ≥ 50%. In our study cohort 56% had a PD-L1 TPS ≥ 50%, resulting in marginally more patients receiving immunotherapy.
Overall representativeness of this study:	
	Based on the above-mentioned characteristics, our study cohort represents a real life cohort of advanced/non-curative locally advanced NSCLC patients without actionable molecular alterations. Exceptions are histology and PD-L1 TPS, which should be included in the interpretation of results. Race/ethnicity is not included as less than 10% of the population in Denmark has a non-western origin.

Table S3. Representativeness of Study Participants
