# Supplementary material for: Circulating Tumor DNA Monitoring Reveals Molecular Progression before Radiologic Progression in a Real-life Cohort of Patients with Advanced Non–small Cell Lung Cancer
Source: Cancer Res Commun. 2022 Oct 13;2(10):1174–87. doi: 10.1158/2767-9764.CRC-22-0258 (PMC10035379; doi:10.1158/2767-9764.CRC-22-0258)
Supplement: Supplementary Table TS4 — Analyses of variables for ctDNA detection [file crc-22-0258-s11.pdf]

Table S4

	**Non-detectable ctDNA (*n*=44)**	**Detectable ctDNA (*n*=88)**	Fisher's Exact test	Logistic regression (sign.)	Odds ratio	95% CI interval
**Variables**	*n* (%)	*n* (%)	*p*			
Adenocarcinoma<br>Non-adenocarc.	38 (86%)<br>6 (14%)	57 (65%)<br>31 (35%)	0.013	0.008	3.868	1.421-10.525
Stage III<br>Stage IV	11 (25%)<br>33 (75%)	12 (14%)<br>76 (86%)	0.143	0.603	1.301	0.483-3.509
Liver metastases	3 (7%)	19 (22%)	0.046	0.055	3.698	0.973-14.058
Adrenal gland metastases	9 (20%)	32 (36%)	0.074	0.048	2.515	1.007-6.284
Pleura fluid	15 (34%)	32 (36%)	0.849	-	-	-
Bone	8 (18%)	24 (27%)	0.288	-	-	-
Brain	5 (11%)	5 (6%)	0.300	-	-	-
Retroperitoneal glands	2 (5%)	10 (11%)	0.336	-	-	-
Standard assay<br>Designed assay	20 (45%)<br>24 (55%)	37 (42%)<br>51 (58%)	0.198	-	-	-

Table S4. Analyses of variables for ctDNA detection
